# Health equity impacts of COVID-19 policies on dementia-relevant community services: A SGBA+ policy scan

**DOI:** 10.1093/geroni/igaa057.3529

**Published:** 2020-12-16

**Authors:** Katie Aubrecht, Rosanne Burke, Jacqueline Gahagan, Laura Dowling, Christine Kelly Mary Jean Hande, Susan Hardie, Janice Keefe

**Affiliations:** 1 St. Francis Xavier University, Antigonish, Nova Scotia, Canada; 2 Mount Saint Vincent University, Halifax, Nova Scotia, Canada; 3 Dalhousie University, Halifax, Nova Scotia, Canada; 4 Evaince (Canadian Centre on Disability Studies), Ottawa, Nova Scotia, Canada; 5 Mount Saint Vincent University, Halifax, Alberta, Canada

## Abstract

This presentation shares the methodology and early findings from a policy scan conducted to understand and assess the impact of COVID-19 policies on dementia care in the community for diverse populations in the province of Nova Scotia, Canada. The scan provided baseline information on: 1) Provincial legislative and regulatory policies related to dementia care in the community; 2) Orders and legislation enacted in response to COVID-19 that potentially impact those policies. Information was obtained from publicly accessible databases and government websites. Searches were also conducted using Google. 135 Acts were collected and reviewed. A specific aim of the scan was to generate knowledge about the impact of these layered policies in the context of a public health crisis from the perspective of local socially and geographically marginalized communities. A Sex and Gender Based Analysis Plus analytical approach was used to assess potential health equity impacts of COVID-19 policies on dementia care in the community. Information was organized using an adapted Health Equity Impact Assessment tool and Systems Health Equity Lens. Strengths and limitations of the approach and tools are discussed.

